# Erythritol production on wheat straw using *Trichoderma reesei*

**DOI:** 10.1186/s13568-014-0034-y

**Published:** 2014-05-29

**Authors:** Birgit Jovanović, Robert L Mach, Astrid R Mach-Aigner

**Affiliations:** 1 Department for Biotechnology and Microbiology, Institute of Chemical Engineering, Vienna University of Technology, Gumpendorfer Str. 1a, A-1060 Wien, Austria

**Keywords:** Erythritol, Erythrose reductase, Trichoderma reesei; Wheat straw; Lignocellulose

## Abstract

We overexpressed the *err1* gene in the *Trichoderma reesei* wild-type and in the cellulase hyperproducing, carbon catabolite derepressed strain Rut-C30 in order to investigate the possibility of producing erythritol with *T. reesei*. Two different promoters were used for *err1* overexpression in both strains, a constitutive (the native pyruvat kinase (*pki*) promoter) and an inducible one (the native *β*-xylosidase (*bxl1*) promoter). The derived recombinant strains were precharacterized by analysis of *err1* transcript formation on D-xylose and xylan. Based on this, one strain of each type was chosen for further investigation for erythritol production in shake flasks and in bioreactor experiments. For the latter, we used wheat straw pretreated by an alkaline organosolve process as lignocellulosic substrate. Shake flask experiments on D-xylose showed increased erythritol formation for both, the wild-type and the Rut-C30 overexpression strain compared to their respective parental strain. Bioreactor cultivations on wheat straw did not increase erythritol formation in the wild-type overexpression strain. However, *err1* overexpression in Rut-C30 led to a clearly higher erythritol formation on wheat straw.

## Introduction

Erythritol is a four-carbon sugar alcohol, which is applied as flavor enhancer, formulation aid, humectant, stabilizer, thickener, and as low-calorie sweetener, of which the latter is the main utilization. Compared with other polyols yielding about 2 cal/g, erythritol yields only up to 0.2 cal/g, which is due to the fact that erythritol does not undergo systemic metabolism in the human body but is excreted unchanged in the urine (Moon et al. [2010]). Additionally, as a small molecule, it is easily absorbed already in the upper intestine and therefore, causes less digestive distress than other sweeteners (Livesey [2001]). Since erythritol is not assimilated by *Streptococcus mutans* it is non-cariogenic. Furthermore it has some favorable physical and chemical properties: it is thermally stable (no decomposition or colorization at 200℃ for 1 h), better crystallizeable than sucrose, and less hygroscopic (Kasumi [1995]). The negative enthalpy of solution leads to a cooling effect when dissolved. The sweetness of erythritol is plain with very weak after-taste. In a 10% (w/v) solution it has 60-80% the sweetness of sucrose. It has a natural occurrence in several foods including beer, sake, wine, soy sauce, water melon, pear, and grape. The tolerance of erythritol by animals and humans was intensively studied (Munro et al. [1998]). No adverse toxicological effects were observed. Also no carcinogenic, mutagenic or teratogenic potential or effects on fertility could be detected. Therefore, erythritol is a sugar substitute with a growing market and optimization of its production remains an issue.

Current biotechnological production of erythritol use osmophilic yeasts like *Aureobasidium* sp., *Trichosporonoides* sp., *Torula* sp., and *Candida magnoliae*. As substrate a highly concentrated glucose (typically 40% (w/v)) solution is applied, which is gained from chemically and enzymatically hydrolyzed wheat- and cornstarch. The hydrolyzed starch serves as carbon source and causes a high osmotic pressure that pushes the yeast to produce the osmolyte erythritol (Moon et al. [2010]). Although these processes reach 40% (w/w) yields of D-glucose to erythritol conversion, they depend on D-glucose as starting material. With regard to (socio)economical issues, starch-derived D-glucose is not a preferable substrate. Therefore, it would be an interesting alternative to use organisms that can utilize non-food, lignocellulosic biomass for the production of erythritol.

In a previous work (Jovanovic et al. [2013]) we characterized the erythrose reductases (Err1) from the filamentous ascomycota *Trichoderma reesei* (telemorph *Hypocrea jecorina* (Kuhls et al. [1996])), *Aspergillus niger*, and *Fusarium graminearium* (telemorph *Gibberella zeae*), which are all very potent degraders of biomass. It turned out that the Err1 of *T. reesei* and *A. niger* showed comparable activities, whereas the Err1 from *F. graminearium* had a considerably lower activity (Jovanovic et al. [2013]). In the present study we focused on the potential of producing erythritol in *T. reesei* from lignocellulosic biomass. The native lignocellulose-degrading enzymes of the fungus have already broad application in industry, i.e. in pulp and paper (Buchert et al. [1998]; Noé et al. [1986]; Welt and Dinus [1995]), food and feed (Galante et al. [1993]; Lanzarini and Pifferi [1989]; Walsh et al. [1993]), and textile industries (Koo et al. [1994], Kumar et al. [1994], Pedersen et al. [1992]) as well as in biofuel production (Hahn-Hägerdal et al. [2006]; Himmel et al. [2007]; Ragauskas et al. [2006]). As such a strong producer of cellulases and hemicellulases (a genome-wide search using the JGI Genome Portal (http://genome.jgipsf.org/Trire2/Trire2.home.html) revealed for *T. reesei* 10 celluloytic and 16 xylanolytic enzyme-encoding genes (Martinez et al. [2008])) it is likely that *T. reesei* is able to grow on cheap biowaste material like wheat straw as the sole carbon source. This is supported by former reports on *T. reesei* capable of growing on lignocellulosic material (Acebal et al. [1986]; Dashtban et al. [2013]).

In this study we used wheat straw that was pretreated by an alkaline organosolve process (Fackler et al. [2012]) to remove the lignin up to a residual concentration of about 1% (w/w), which makes the cellulose and hemicellulose more easily accessible for the fungus. We investigated a *T. reesei* wild-type strain and the strain Rut-C30. Rut-30 is a cellulase hyperproducing, carbon catabolite derepressed mutant (Montenecourt and Eveleigh [1979]), which is the parental strain of most industrially used *T. reesei* strains (Peterson and Nevalainen [2012]; Derntl et al. [2013]). In both strains the *err1* gene was overexpressed using either the native, constitutive promoter from the pyruvate kinase encoding gene (*pki*) or the native, inducible promoter from the *β*-xylosidase 1 encoding gene (*bxl1*). The overexpression strains were screened for enhanced *err1* transcript formation and the best ones where then cultivated on D-xylose and wheat straw for investigating their erythritol production capacity.

## Materials and methods

### Strains and cultivation conditions

The *T. reesei* strains QM6a *Δtmus53* (Steiger et al. [2011]) and Rut-C30 (ATCC 56765), which was derived from the wild-type strain QM6a by one UV-light and two N-methyl-N’-nitro-N-nitrosoguanidine mutation steps (Montenecourt and Eveleigh [1979]), were maintained on 3% malt extract (MEX) agar. The recombinant *T. reesei* strains QPEC1, QBEC2, RPEC1, and RBEC2 generated during this study, were maintained on MEX agar containing 250 *μ*l/l hygromycin B (Merck, Darmstadt, Germany).

Purification of transformed strains by streak out of spores was done on MEX agar containing 250 *μ*l/l hygromycin B and 500 *μ*l/l IGEPAL®; CA-630 (Sigma-Aldrich, St. Louis, MO, USA).

Cultivation in shake flasks was performed in 250-ml-Erlenmeyer flasks containing 50 ml Mandels-Andreotti (MA) medium (Mandels [1985]) supplemented with 1% (w/v) D-xylose or 1% (w/v) birch-wood xylan. For inoculation 10^9^ conidia per liter were used. Growth conditions were pH 5, 30℃, and 160 rpm shaking rate. Mycelia and supernatant were seperated by filtration. For short-term storage, harvested mycelia were shock-frozen and kept in liquid nitrogen, supernatants were kept at -20℃.

### Plasmid construction

The *err1* gene and the promoter region of *bxl1* (1.5 kbp upstream *bxl1*, p*bxl1*) from *T. reesei* were amplified from cDNA, which was generated as described below in the according section. Primers were used to introduce restriction sites adjacent to the gene. Primer sequences are given in Table 1. The PCR product was subcloned into pJET-1.2 (Thermo Scientific, Waltham, MA, USA), using chemically competent *Escherichia coli* TOP 10 (Invitrogen, Life Technologies Ltd, Paisley, UK) for plasmid replication.

**Table 1:** Oligonucleotides used during the study

**Name**	**Sequence (5’ – 3’)**	**Usage**
pbxl1_SalI__EcoRI_f	ATATAGTCGACGAATTCAGCTTGTCTGCCTTGATTACCATCC	Vector construction
pbxl1_XbaI_r	ATATATCTAGATGCGTCCGGCTGTCCTTC	Vector construction
err1_XbaI_f	ATATATCTAGAATGTCTTCCGGAAGGACC	Vector construction
err1_Nsi_r	TATATATGCATTTACAGCTTGATGACAGCAGTG	Vector construction
ppki_f	GCACGCATCGCCTTATCGTC	PCR test
qerr1_f	CTTTACCATTGAGCACCTCGACG	RT-qPCR
qerr1_r	GGTCTTGCCCTGCTTCTTGG	RT-qPCR
qact1_f	TGAGAGCGGTGGTATCCACG	RT-qPCR
qact1_r	GGTACCACCAGACATGACAATGTTG	RT-qPCR
qsar1_f	TGGATCGTCAACTGGTTCTACGA	RT-qPCR
qsar1_r	GCATGTGTAGCAACGTGGTCTTT	RT-qPCR

For the construction of pBJ-PEC1 the vector pRLM _*ex*30_ (Mach et al. [1994]), which contains the *hph* gene flanked by the *pki* promoter (p*pki*) and the *cbh2* terminator, was used. The *hph* gene was removed by *Nsi*I/*Xba*I digestion and subsequently, *err1* that was excised from pJET-1.2 also by *Nsi*I/*Xba*I digestion, was inserted.

For the construction of pBJ-BEC2 p*pki* was excised from pBJ-PEC1 with *Xho*I/*Xba*I digestion and replaced by p*bxl1*, excised from pJET-1.2 with *Sal*I/*Xba*I digestion.

### Protoplast transformation

For QM6a *Δtmus53* protoplast transformation was performed as described in (Gruber et al. [1990]). 5 g of either pBJ-PEC1 or pBJ-BEC2 and 1 *μ*g pAN7, which confers hygromycin B resistance (Punt et al. [1987]), were co-transformed into the fungal genome.

### Biolistic transformation

Rut-C30 was transformed with the Biolistic®; PDS-1000/He Particle Delivery System (Bio-Rad Laboratories, Hercules, CA, USA) according to a modified protocol originally described in (Te’o et al. [2002]). 5 *μ*g of either pBJ-PEC1 or pBJ-BEC2 and 1 *μ*g pAN7, which confers hygromycin B resistance (Punt et al. ([Punt et al. 1987])), were co-transformed into the fungal genome.

### DNA isolation

Fungal genomic DNA was isolated by phenol-chloroform extraction, using a FastPrep®;-24 (MP Biomedicals, Santa Ana, CA, USA) for cell disruption. About 100 mg of mycelia was transferred to 400 *μ*l DNA extraction buffer (0.1 M Tris-HCl pH 8.0, 1.2 M NaCl, 5 mM EDTA) and grounded with glass beads (0.37 g 0.01 0.1 mm, 0.25 g 1 mm, 1 piece 3 mm) using the FastPrep. Afterwards, the mixture was immediately put on 65℃, supplemented with 9 *μ*M RNase A, and incubated for 30 min. Then 200 *μ*l of phenol (pH 7.9) and 200 *μ*l of a chloroform-isoamyl alcohol-mixture (25:1) were added, with vigorous mixing following each addition. Phases were separated by centrifugation (12000 g, 10 min, 4℃) and the aqueous phase was transferred into a new vial. DNA was precipitated by addition of the 0.7-fold volume of isopropanol to the aqueous phase. After 20 min incubation at room temperature (RT) the DNA was separated by centrifugation (20000 g, 20 min, 4℃) and washed with 500 *μ*l ethanol (70%). The air-dried DNA pellet was solubilised in 50 *μ*l Tris-HCl (10 mM, pH 7.5) at 60℃.

### RNA isolation and cDNA synthesis

RNA extraction from fungal mycelia was performed with peqGOLD TriFast™ (peqlab, Erlangen, Germany) according to the manufacturer’s procedure, using a FastPrep®;-24 (MP Biomedicals) for cell disruption. RNA quantity and quality were determined with a NanoDrop 1000 (Thermo Scientific). A 260 nm/280 nm ratio of at least 1.8 was stipulated for further sample processing. cDNA synthesis was performed with RevertAid™H Minus First Strand cDNA Synthesis Kit (Thermo Scientific) according to the manufacturer’s procedure using 0.5 *μ*g of RNA.

### Transcript analysis

RT-qPCR analysis was performed in a Rotor-Gene Q cycler (Qiagen, Hilden, Germany). The qPCR amplification mixture had a total volume of 15 *μ*l, containing 7.5 *μ*l 2x IQ SYBR Green Supermix (Bio-Rad Laboratories), 100 nM forward and reverse primer, and 2 *μ*l cDNA (diluted 1:100). Primer sequences are given in Table 1. As reference genes *act1* and *sar1* were used (Steiger et al. [2010]). All reactions were performed in triplicates. For each gene a no-template control and a no-amplification control (0.01% SDS added to the reaction mixture) was included in each run. The cycling conditions for *act1* and *err1* comprised 3 min initial denaturation and polymerase activation at 95℃, followed by 40 cycles of 15 s at 95℃, 15 s at 59℃ and 15 s at 72 s. For *sar1* different cycling conditions were applied: 3 min initial denaturation and polymerase activation at 95℃, followed by 40 cycles of 15 s at 95℃, and 120 s at 64 s. PCR efficiency was calculated from the Rotor-Gene Q software. Relative expression levels were calculated using the equation

(1) $$\text{relative}\;\text{transcript}\;\text{ratio}=E_{r}^{C_{r}}\cdot E_{t}^{-C_{t}}\cdot E_{r_{0}}^{-C_{r_{0}}}\cdot E_{t_{0}}^{C_{t_{0}}},$$

where *E* is cycling efficiency, *C* is the threshold cycling number, *r* is the reference gene, *t* the target gene and a 0 marks the sample which is used as the reference (Pfaffl [2001]).

### Probe preparation for Southern blot analysis

For the probe preparation 500 ng of *err1* cDNA, 5 *μ*l 10x Klenow buffer (Thermo Scientific), and 6.5 *μ*l 100 *μ*M random hexamer primer (Thermo Scientific) were filled up with double distilled water (ddH_2_O) to a final volume of 39 *μ*l and incubated at 95℃ for 5-10 min. The reaction mixture was put on ice and 5 *μ*l Biotin PCR Labeling Mix (Jena Bioscience, Jena, Germany) and 1 *μ*l Klenow fragment exo- (Thermo Scientific) were added. The mixture was filled up with ddH_2_O to a final volume of 50 *μ*l and incubated at 37℃ for 24 h. For DNA precipitation 10 *μ*l LiCl (4 M) and 200 *μ*l ethanol (96%) were added. After incubation at RT for 15 min, at ice for 15 min, and at -20℃ for 1 h, the DNA was separated by centrifugation with 20000 g at 4℃ for 30 min. The pellet was washed with 500 *μ*l ethanol (70%), followed by centrifugation with 20000 g at 4℃ for 10 min. After drying the pellet at 50℃ for about 10 min, it was dissolved in 100 *μ*l ddH_2_O. The quality of the probe was tested by agarose gel electrophoresis, and the concentration was determined with the NanoDrop 1000 (Thermo Scientific).

### Southern blot analysis

For the Southern blot 15 *μ*g chromosomal DNA of each strain used in this study was digested in a triple digestion with 5 *μ*l of each *Nde*I, *Sal*I, and *Bgl*II (each 10 U/ *μ*l, Thermo Scientific), using 10x Buffer O (Thermo Scientific). The reaction mixtures were filled up with ddH_2_O to a final volume of 100 *μ*l and then split into 20 *μ*l aliquots for digestion at 37℃ over night (o/n). After digestion, samples were incubated at 70℃ for 20 min and the completion of digestion controlled by agarose gel electrophoresis. Digestion aliquots of each sample were pooled and concentrated to a final volume of 10 - 20 *μ*l and applied to a 1% agarose gel. As length standard 5 *μ*l Gene Ruler 1 kb DNA Ladder (Thermo Scientific) was used. Using the Mini-Sub®; Cell system (Bio-Rad Laboratories) the gel was run at 80 V for 1 h in TAE buffer, and afterwards incubated in 0.4 M NaOH and 0.6 M NaCl, and then in 0.5 M Tris (pH 7.5) and 1.5 M NaCl for 30 min each. The DNA was transferred to a Biodyne B membrane (Pall Corporation, Port Washington, NY, USA) by a capillary blot with 10x saline-sodium citrate (SSC) buffer (1.5 M NaCl, 0.15 M sodium citrate, pH 7.2) o/n. After blotting, the membrane was incubated in 0.4 M NaOH, and then in 0.2 M Tris (pH 7.5) for 1 min each. Cross-linking was performed with a GS Gene Linker UV chamber (Bio-Rad Laboratories) using program C3 and 150 mJoule on the wet membrane. For pre-hybridization, the membrane was incubated at 65℃ for 3 h in 20 ml Southern blot hybridization buffer (25% (v/v) 20x SSC, 10% (v/v) 50x Denhardt’s solution, 0.2% (v/v) EDTA (0.5 M, pH 8.0), 0.05 M NaH_2_PO_4_, 0.1% (w/v) SDS, 0.5% (w/v) BSA), supplemented with 100 *μ*g/ml single stranded salmon sperm DNA and freshly denaturated (10 min at 95℃) probe. For hybridization, the membrane was incubated at 65℃ o/n in 10 ml Southern blot hybridization buffer. The membrane was washed twice at RT for 5 min in 50 ml 2x SSC supplemented with 0.1% (w/v) SDS, followed by washing twice at 65℃ for 15 min with 50 ml 0.1x SSC, supplemented with 0.1% (w/v) SDS. After incubation at RT for 10 min in Southern blot blocking solution (125 mM NaCl, 17 mM Na_2_HPO_4_, 8 mM NaH_2_PO_4_, 0.5% (w/v) SDS, pH 7.2), the membrane was incubated light-protected at RT for 30 min in Southern blot blocking solution supplemented with 1 *μ*g/ml Dylight 650-labeled Streptavidin (Thermo Scientific). The membrane was washed 4 times light-protected at RT for 10 min in 50 ml 1:10 diluted Southern blocking solution and then scanned with Typhoon FLA 9500 (GE Healthcare Life Sciences, Buckinghamshire, England) set for Alexa Fluor 647 at 1000 V.

### Cultivation in bioreactors

Cultivation was performed in 2-l-bench top bioreactors (Bioengineering AG, Wald, Swiss), containing 1.3 l fermentation medium ((NH_4_)_2_SO_4_ 3.50 g/l, KH_2_PO_4_ 5.00 g/l, MgSO_4_.7H_2_O 1.25 g/l, NaCl 0.625 g/l, peptone from Casein 1.25 g/l, Tween®; 80 0.625 g/l), supplemented with 1.5 ml/l trace element solution (FeSO_4_.7H_2_O 0.90 mM, MnSO_4_.H_2_O 0.50 mM, ZnSO_4_.7H_2_O 0.24 mM, CaCl_2_.7H_2_O 0.68 mM), 1.7% (w/v) wheat straw (pretreated by an alkaline organosolv process for lignin removal (Fackler et al. [2012]) (Annikki, Graz, Austria)), and Antifoam Y-30 Emulsion (1 ml/bioreactor). For inoculation 10^9^ conidia per liter were used. Agitation rate was 500 rpm, temperature was 28℃, and aeration rate was 0.5 vvm.

### GC analysis

Mycelia from shake flask cultures were ground under liquid nitrogen. The powder was suspended in 3 ml distilled water and sonicated using a Sonifier®; 250 Cell Disruptor (Branson, Danbury, CT, USA) (power 70%, duty cycle 40%, power for 3 min, on ice). Insoluble compounds were separated by centrifugation (20000 g, 10 min, 4℃), the clear supernatant was used for further processing.

Supernatants from shake flask cultures were used directly for further processing.

For samples from cultivation in bioreactors 30 ml of the whole cultivation broth were first mechanically disrupted with a potter for 1 min, then sonicated, and afterwards centrifuged as described above for mycelia from shake flask cultures.

Sample preparation for GC was done in triplicates as follows: 300 *μ*l of the clear supernatant (prepared as described above), supplemented with 10 ng myo-inositol as internal standard, was gently mixed with 1.2 ml ethanol (96%) and incubated for 30 min at RT for protein precipitation. The precipitate was separated by centrifugation (20000 g, 10 min, 4℃). Samples were dried under vacuum and thereafter silylated (50 *μ*l pyridine, 250 *μ*l hexamethyldisilazane, 120 *μ*l trimethylsilyl chloride). For quantitative erythritol determination a GC equipment (Agilent Technologies, Santa Clara, CA, USA) with a HP-5-column (30 m, inner diameter 0.32 mm, film 0.26 *μ*m) (Agilent) was used. The mobile phase consisted of helium with a flow of 1.4 l/min, the column temperature was as follows: 150℃ for 1 min, ramping 150 220℃ (*Δ*T 4℃/min), ramping 220 - 320℃ (*Δ*T 20℃/min), 320℃ for 6.5 min. Detection was performed with FID at 300℃. The retention times were determined using pure standard substances.

### Sodium hydroxide soluble protein (SSP)

2 ml cultivation broth were centrifuged at 20000 g for 10 min at 4℃. The supernatant was discarded and the pellet resuspended in 3 ml 0.1 M NaOH before sonication with a Sonifier®; 250 Cell Disruptor (Branson) (power 70%, duty cycle 40%, power 20 s, pause 40 s, 10 cycles, on ice). The sonicated samples were incubated for 3 h at RT. After centrifugation (20000 g, 10 min, 4℃) the supernatant was used to determine protein concentration with a Bradford assay. Therefore, 20 *μ*l diluted sample (1:10 - 1:100) were added to 1 ml 1:5 diluted Bradford Reagens (Bio-Rad Laboratories) and incubated for exactly 10 min at RT before measuring the absorption on a V-630 UV-Vis spectrophotometer (Jasco, Tokio, Japan) at 595 nm. As standard bovine serum albumin in concentrations from 10 - 100 *μ*g/ml was used.

## Results

### Characterization of *err1* overexpression strains

**Figure 1:**
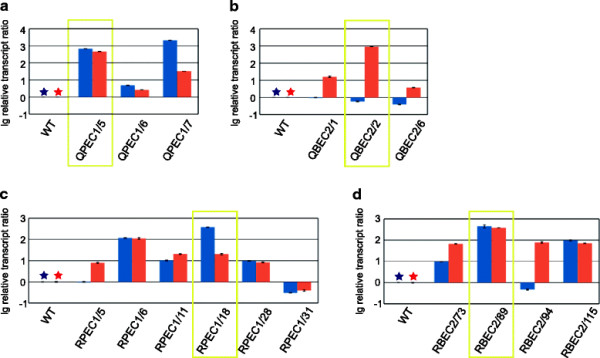
**Transcript analysis of*err1* in parental and recombinant*T. reesei* strains.** The *T. reesei* wild-type strain (WT) and preselected recombinant strains derived from transformation of the wild-type **(a, b)** or of Rut-C30 **(c, d)**, which are expressing *err1* either under the constitutive *pki* promoter **(a, c)** or under the inducible *bxl1* promoter **(b, d)**, respectively, were cultivated in shake flasks on D-xylose (blue bars) for 30 h (wild-type) or 72 h (Rut-C30) and on birch-wood xylan (red bars) for 48 h (wild-type) or 72 h (Rut-C30). Strains chosen for further experiments are framed in yellow. The transcript analysis was performed by qPCR using *sar1* and *act1* as genes for data normalization and levels always refer to the wild-type strain on the respective carbon source (indicated by a blue and red asterisk). Results are given as relative transcript ratios in logarithmic scale (lg). The values are means from three measurements. Error bars indicate standard deviations. Biological experiments (cultivations) were performed in duplicates.

**Figure 2:**
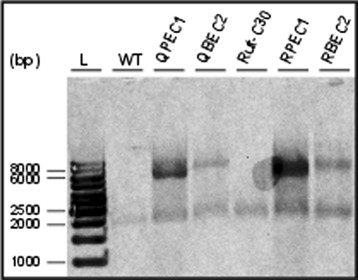
**Southern blot analysis of parental and*err1* overexpression*T. reesei* strains.** On an agarose gel *Nde*I/*Sal*I/*Bgl*II-digested DNA from the wild-type (WT) strain bearing the native *err1* (2340 bp), the thereof derived *err1* overexpression strains QPEC1 (containing the native *err1* (2340 bp) and n+1 inserted fragments (5410 bp)) and QBEC2 (containing the native *err1* (2340 bp) and n+1 inserted fragments (6626 bp)), Rut-C30 bearing the native *err1* (2340 bp), and the thereof derived *err1* overexpression strains RPEC1 (containing the native *err1* (2340 bp) and n+1 inserted fragments (5410 bp)) and RBEC2 (containing the native *err1* (2340 bp) and n+1 inserted fragments (6626 bp)) was separated. n means the band intensity in relation to the native *err1*-containing band. A 1 kb DNA ladder (L) was used for estimation of DNA fragment size; indicated sizes are given in bp. As probe a biotin-labeled fragment containing the structural *err1* gene was used. For visualization Dylight 650-labeled streptavidin was applied and the membrane was scanned with a Typhoon FLA 9500.

Protoplast transformation of the wild-type strain with the plasmid pBJ-PEC1, introducing *err1* under the constitutive *pki* promoter of *T. reesei*, yielded 8 recombinant strains (named QPEC1-#). With the plasmid pBJ-BEC2, introducing *err1* under the inducible *bxl1* promoter of *T. reesei*, 3 recombinant strains (named QBEC2-#) were received. Biolistic transformation of Rut-C30 with the plasmid pBJ-PEC1 yielded 12 recombinant strains (named RPEC1-#), and with the plasmid pBJ-BEC2 20 recombinant strains (named RBEC2-#) were obtained. Stable insertion of the plasmid into the fungal genome was confirmed by isolation of genomic DNA and a following PCR amplifying a fragment including the introduced promoter and *err1*. After two rounds of spore streak outs, 3-7 recombinant strains of all four types were chosen for further characterization according to their growth. The selected recombinant strains were cultivated in shake flasks on D-xylose as well as birch-wood xylan followed by transcript analysis of *err1*. From each type, the strain with the highest transcript rate was chosen for further characterization (Figure 1). From now on strains were termed QPEC1, QBEC2, RPEC1, and RBEC2, respectively. A determination of the copy number of the newly introduced *err1* in the four finally selected recombinant strains was performed by Southern blot analysis (Figure 2). Ectopic *in tandem* integration, which is the most common in *T. reesei* (Mach and Zeilinger [1998]), was observed in all four strains. For QPEC1 and RPEC1 more than 5 additional copies were estimated, for QBEC2 and RBEC2 1-2 additional copies were estimated.

### Increased production of erythritol on D-xylose

**Figure 3:**
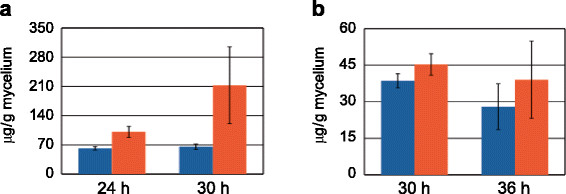
**Erythritol production on D-xylose.** The *T. reesei***(a)** wild-type strain (blue bars) and the thereof derived *err1* overexpression strain QPEC1 (red bars) as well as **(b)** Rut-C30 (blue bars) and the thereof derived *err1* overexpression strain RPEC1 (red bars) were cultivated in shake flasks on D-xylose. Samples were taken after the indicated time and erythritol concentration was determined by GC-analysis from cell free extracts. Biological experiments (cultivations) were performed in duplicates. Standard deviations were obtained from two biological duplicates and measurements in triplicates each.

In order to get first insight in the native erythritol formation in the parental strains and the effect of the *err1* overexpression, the strains QPEC1 and RPEC1, as well as their respective parental strains, were cultivated in shake flasks. For this first experiment D-xylose was used as carbon source as all strains grow well on this carbon source, and on the other hand as the monomer of the xylan-backbone it is a main component of lignocellulose, which is aimed to be used finally. Samples were taken after biomass formation was observed and analyzed by gas chromatography (GC) for erythritol production. Separate analysis of the supernatant and the mycelia revealed that no erythritol could be found in the supernatant. The erythritol concentrations detected in the mycelia are presented in Figure 3. For the wild-type and QPEC1 we could demonstrate, that the overexpression strain contained clearly more erythritol than the parental strain, with an increase of 1.6-fold (24 h) and 3.2-fold (30 h) (Figure 3a). For Rut-C30 and RPEC1 the increase of intracellular erythritol concentration in the *err1* overexpression strain are not that explicit compared to the wild-type and to QPEC1 (Figure 3b). After 30 h and 36 h the increase in the recombinant strain is 1.2-fold and 1.4-fold, respectively, compared to the parental strain. Compared with the wild-type, both Rut-C30 and RPEC1 contained slightly less erythritol. However, this observation was considered as a preliminary result because the advantages of using Rut-C30 are not necessarily that pronounced on D-xylose than on a lignocellulosic substrate, which finally should be used according to the aim of this study.

### Erythritol formation by the wild-type and its *err1* overexpression strains on pretreated wheat straw

**Figure 4:**
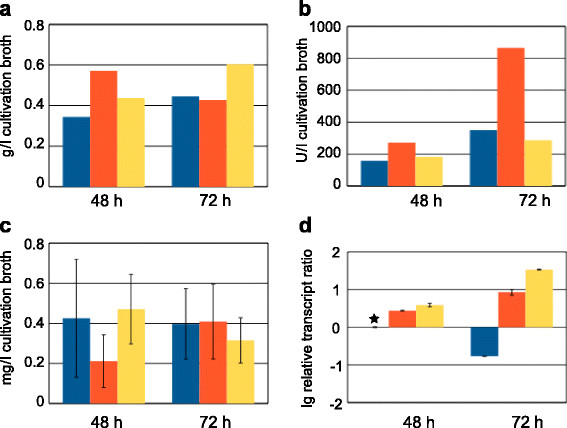
**Cultivation of the wild-type and*err1* overexpression strains on wheat straw.** The *T. reesei* wild-type strain (blue bars), and the *err1* overexpression strains QPEC1 (red bars) and QBEC2 (yellow bars) were cultivated in bench-top bioreactors on pretreated wheat straw. Samples were taken after 48 and 72 h. **(a)** Sodium soluble protein concentration (given in g/l cultivation broth) was measured in triplicates in cultivation broth samples after cell disruption to indicate biomass formation. Standard deviations were below 5%. **(b)** Xylanase activity (given in U/l cultivation broth) was measured in triplicates in the cultivation supernatants. Standard deviations were below 5%. **(c)** Erythritol concentration (given in mg/l cultivation broth) was measured in triplicates by GC in cultivation broth samples after cell disruption. Error bars indicate standard deviations. **(d)** Transcript analysis of *err1* (given as relative transcript ratio in logarithmic scale (lg)) was performed by qPCR in triplicates using *sar1* and *act1* as genes for data normalization and levels always refer to the wild-type strain cultivated for 48 h (as indicated by an asterisk). Error bars indicate standard deviations. Biological experiments (cultivations) were performed in duplicates.

Experiments to investigate the growth ability on pretreated wheat straw and corresponding erythritol production on this substrate were performed by cultivation in a bioreactor starting with the wild-type strain and its respective *err1* overexpression strains, QPEC1 and QBEC2. All three strains were able to grow on wheat straw as sole carbon source, even if inoculated directly with conidia and not with pregrown fungal mycelium. Microscopic analysis of samples taken 8 h after inoculation already showed a high germination rate. Further microscopic samples taken during the fermentation process showed good mycelial growth, strongly branched hyphae, and disappearance of the straw, which is due to enzymatic degradation by the fungus. Samples for investigation of erythritol production were taken 48 h and 72 h after inoculation. Since the cultivation broth contained aside from the mycelia also wheat straw as insoluble compound, it was not possible to separate the mycelia for analysis. Therefore, the whole samples were analyzed for erythritol content. Sodium hydroxide soluble protein (SSP) was determined and was used as an indicator for the biomass concentration. From the SSP one can conclude that the strains have a similar growth behavior (Figure 4a). The xylanase activity was similar in the wild-type and in QBEC2, but clearly increased in QPEC1 after 72 h (Figure 4b). In contrast to the results from the shake flask experiments on D-xylose, no increase in production of erythritol in the recombinant strains could be found by GC analysis (Figure 4c), even though transcript analysis of *err1* showed a slight increase in the recombinant strains after 48 h, and an even more pronounced one after 72 h (Figure 4d). Summarizing, these strains can grow on wheat straw and metabolize derived monosaccharides to erythritol. However, overexpression of *err1* did not enhance erythritol formation on wheat straw.

### Erythritol formation by Rut-C30 and its *err1* overexpression strains on pretreated wheat straw

**Figure 5:**
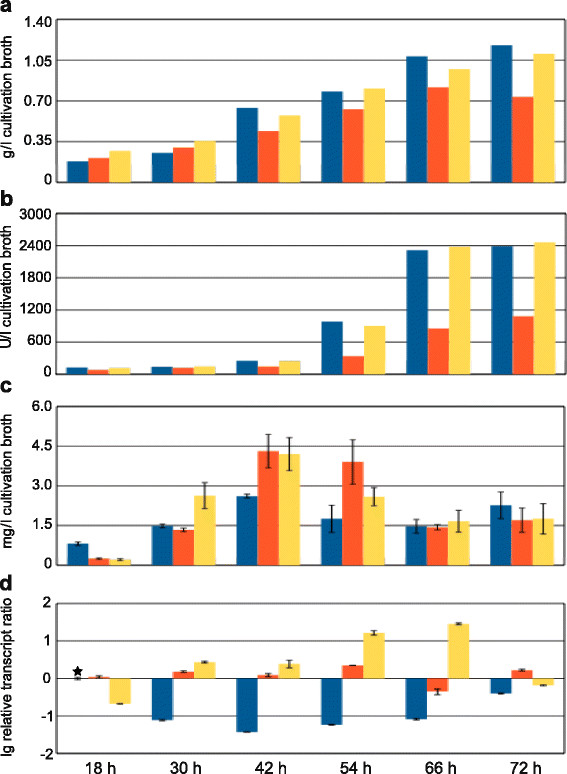
**Cultivation of Rut-C30 and*err1* overexpression strains on wheat straw.** Rut-C30 (blue bars), and the *err1* overexpression strains RPEC1 (red bars) and RBEC2 (yellow bars) were cultivated in bench-top bioreactors on pretreated wheat straw. Samples were taken after 18, 30, 42, 54, 66, and 72 h. **(a)** Sodium soluble protein concentration (given in g/l cultivation broth) was measured in triplicates in cultivation broth samples after cell disruption to indicate biomass formation. Standard deviations were below 5%. **(b)** Xylanase activity (given in U/l cultivation broth) was measured in triplicates in the cultivation supernatants. Standard deviations were below 5%. **(c)** Erythritol concentration (given in mg/l cultivation broth) was measured in triplicates by GC in cultivation broth samples after cell disruption. Error bars indicate standard deviations. **(d)** Transcript analysis of *err1* (given as relative transcript ratio in logarithmic scale (lg)) was performed by qPCR in triplicates using *sar1* and *act1* as genes for data normalization and levels always refer to Rut-C30 cultivated for 18 h (as indicated by an asterisk). Error bars indicate standard deviations. Biological experiments (cultivations) were performed in duplicates.

As a cellulase hyperproducing strain, Rut-C30 can be expected to better utilize lignocellulosic substrates compared to the wild-type strain. Indeed, an analog experiment to the one described above, using Rut-C30 and RPEC1 showed more promising results as increased erythritol production in the overexpression strain was observed (Additional file 1). Consequently, a more extensive study drawing samples every 12 h, starting 18 h after inoculation, was conducted with these strains again cultivated in a bioreactor on pretreated wheat straw. The SSP indicated an similar growth behavior for all strains, whereupon RPEC1 after 42 h slightly dropped behind the others (Figure 5a). The same pattern could be observed even more clearly for the xylanase activities (Figure 5b). The course of erythritol concentration is depicted in Figure 5c. One can observe that the parental strain Rut-C30 started slightly faster with erythritol formation. All strains reached their maximum erythritol production after 42 h, whereupon the *err1* overexpression strains showed increased formation compared to their parental strain. Even though RPEC1 and RBEC2 shared nearly the same maximum erythritol concentration, they differed in their time course of production. Erythritol formation by RBEC2 rose faster in the beginning, but also dropped faster after having reached the maximum. After 66 h the erythritol concentration dropped for all three strains to a nearly equal level, so it seems that the overexpression of *err1* does not only boost the formation of erythritol but might also trigger the erythritol consumption of this storage compound when conditions (e.g. carbon source availability) become less favorable. It should also be noticed that the amount of erythritol produced by the recombinant strains was about 10-fold higher compared to the wild-type at the peak of production. The transcript analysis showed constant expression of *err1* for RPEC1 and an increasing expression for RBEC2, which is in good accordance with the type of promoters used. The expression of *err1* in the parental strain first decreased until it reaches a minimum at 42 h after inoculation. Afterwards, it slightly reincreased, but always remained lower than in the overexpression strains (Figure 5d).

## Discussion

The by an alkaline organosolve process pretreated wheat straw (Fackler et al. [2012]) used in our experiments, turned out to be a very well utilizeable substrate for *T. reesei* cultivation. In contrary to other pretreatment processes, this method does not require any chemicals or catalysts that subsequently inhibit fungal growth. The alcohol, which is used in the process as organic solvent, can be sufficiently removed by washing. The achieved removal of lignin (up to a residual share of 1%) (Fackler et al. [2012]) makes the utilizeable cellulose and hemicellulose enough accessible for the fungus so that even direct inoculation with conidia was possible with this substrate as sole carbon source.

The comparison of the recombinant strains with their respective parentals showed that the overexpression of *err1* was successful an led to an increase in erythritol formation. In case of the wild-type and its recombinant strains this effect was more pronounced in shake flask cultivations on D-xylose, whereas Rut-C30 and its recombinant strains yielded better results in the bioreactor cultivation on pretreated wheat straw. Not only the relative increase of erythritol concentration in the recombinant strains compared to the parental strain was higher, but also the total amount of erythritol produced was about 10-fold increased compared to the wild-type and its recombinant strains. This observation can be explained by the fact that Rut-C30 is a cellulase hyperproducing, carbon catabolite derepressed strain (Montenecourt and Eveleigh [1979]), which makes it very likely that it better utilizes a complex substrate like wheat straw. This assumption is supported by the observed increased biomass formation and enhanced xylanase activity produced. Concerning the promoters used, the constitutive *pki* promoter seems to be favorable, since the erythritol production peak was slightly higher and this high level remained for a longer period (54 h). It should be mentioned that an even higher maximum might occur between the samples taken. However, cultivation time turned out to be an important factor for the erythritol formation, since after the peak of production, the erythritol concentration drops about as fast as it rises in the beginning. Accordingly, the elimination of the back reaction can be considered as one of the main targets of further strain improvement. Since we found that in *T. reesei* erythritol is not exported to the media, but accumulated in the cell, presumably, the most efficient way to prevent the back reaction would be to force the fungus to secrete the erythritol. This strategy would also be favorable in consideration of the osmotic balance of the cell. Taking into account that in case of erythritol production methods using yeasts, erythritol can be found in the supernatant (see e.g. Ryu et al. [2000]; Rymowicz et al. [2009]; Sawada et al. [2009]), in yeasts must exist a transport system for erythritol that probably can be introduced into *T. reesei*. Another strategy to improve erythritol formation could be to reduce the accumulation of other polyols. This would on the one hand provide additional starting material for the erythritol production, and at the same time it would prevent an additional rise of the intracellular osmotic pressure by these substances.

**Figure 6:**
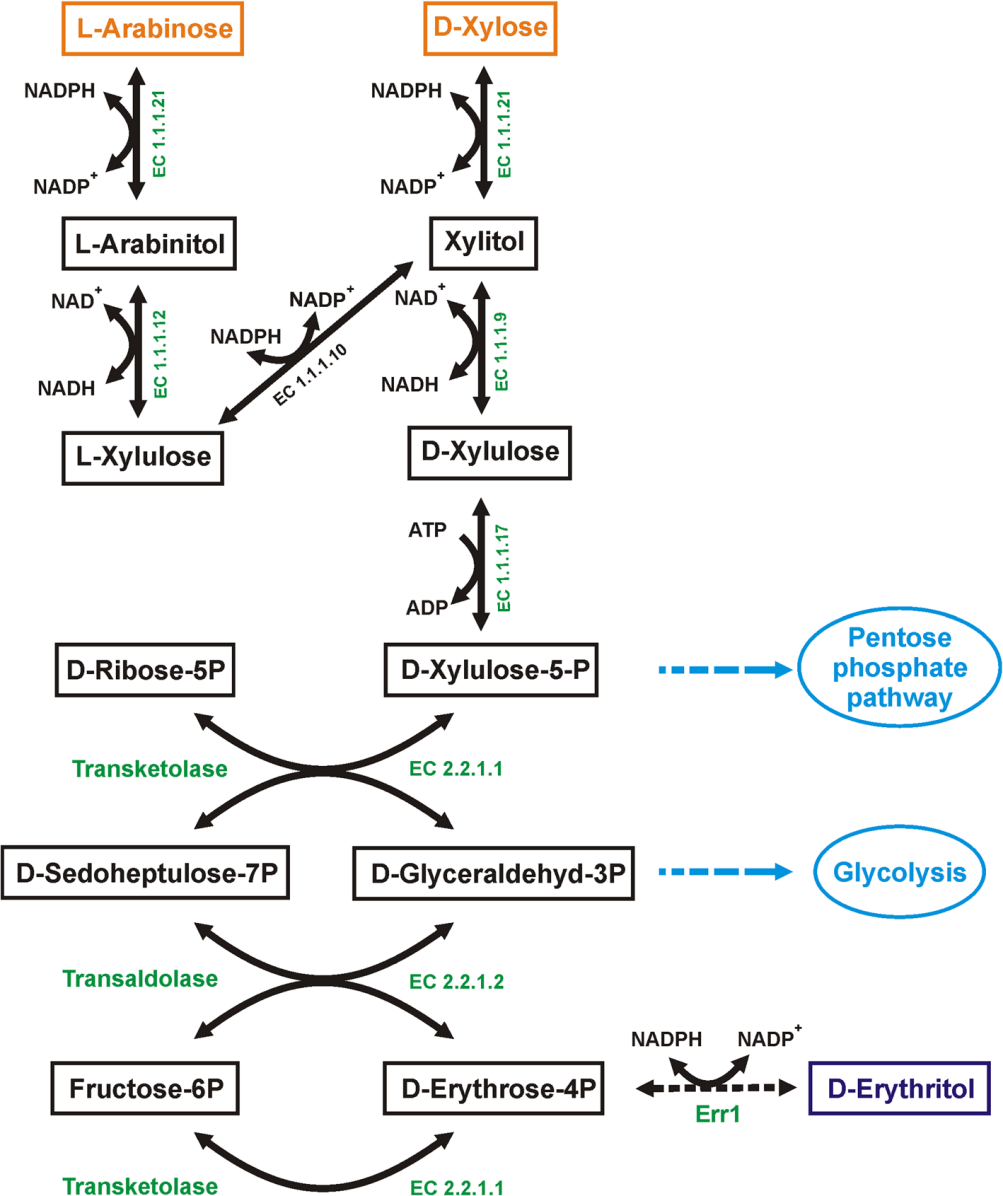
**Schematic drawing of metabolic pathways of pentoses and erythritol in*T. reesei*.** Metabolites are given in boxes. Monomeric sugars derived from hydrolytic lignocellulose degradation by *T. reesei* are given in orange. The target substance, erythritol, is given in purple. Enzyme names and EC numbers are given in green. Adjacent pathways are indicated in blue. Dashed arrows indicate (possible) involvement of more than one enzyme.

GC analysis of the cultivation broth of Rut-C30 and its recombinant strains grown on wheat straw revealed especially a high accumulation of arabinitol, but also considerable amounts of xylitol (highest concentrations measured were 696 mg arabinitol and 63 mg xylitol per liter fermentation broth). Both substances are metabolites in the interconversion of the pentoses derived from lignocellulose degradation (i.e. L-arabinose, D-xylose) (Figure 6). Overexpression of the L-arabinitol dehydrogenase and the D-xylulose reductase in *T. reesei* might help here to enforce the flux of these two major substrates into the pentose phosphate pathway (PPP) and thus enhance erythritol formation, which is a side product of the PPP. Even if the amounts of erythritol produced by now in *T. reesei* (approx. 5 mg/l) do not reach the current production standards with yeasts, it must be taken into consideration, that these yeast strains are highly mutagenized, and subsequently selected for high erythritol production for many years, and production conditions were optimized for decades now. For example, in 1999 for *C. magnolia* 6.9 g/l erythritol formation was reported for the wild-type, while with mutant strains concentrations of up to 25 g/l were obtained (Yang et al. [1999]). Years later further process optimization led to a maximum final erythritol concentration of 200 g/l if fed with 70% (w/v) glucose supplemented with yeast extract (Koh et al. [2003]). As D-glucose became the almost exclusively used substrate for erythritol production, usually the D-glucose to erythritol conversion (in % (w/w)) is reported. Recently, for the *C. magnolia* NCIM 3470 mutant R23 a yield of 31.1% was reported (Savergave et al. [2011]), while with *Trichosporonoides megachiliensis* SN-G42 a conversion of 47% can be achieved (Sawada et al. [2009]). However, the conversion of D-glucose to erythritol can not easily directly be compared to erythritol production from wheat straw due to the very different nature of these substrates. Anyway, the aim of this study was to provide proof-of-concept for the synthesis of erythritol from wheat straw and additional metabolic engineering as described above. Subsequent strain screening might lead to competitive production levels in biomass-degrading fungi like *T. reesei*, with the advantage of using cheap and sustainable substrates.

Concluding this, in the present study we demonstrated that the production of erythritol on the renewable, non-food substrate wheat straw, using *T. reesei* is possible. The alkaline organosolve pretreatment process used for the wheat straw is compatible for subsequent fungal growth and provides an easily utilizeable substrate. Moreover, strain modification by overexpression of *err1* led to increased erythritol formation on this substrate.

## Competing interests

A European patent entitled ‘Method for the production of erythritol‘ (no. EP20100183799, 5.4.2012) (Mach and Mach-Aigner [2012]) was issued.

## Additional file

## Supplementary Material

Additional file 1 **Figure S1.** Cultivation of Rut-C30 and an *err1* overexpression strain on wheat straw. The Rut-C30 (blue bars) and the *err1* overexpression strain RPEC1 (red bars) were cultivated in bench-top bioreactors on pre-treated wheat straw. Samples were taken after 48 and 72 hours. (a) Sodium soluble protein concentration (given in g/l cultivation broth) was measured in triplicates in cultivation broth samples after cell disruption to indicate biomass formation. Standard deviations were below 5%. (b) Xylanase activity (given in U/l cultivation broth) was measured in triplicates in the cultivation supernatants. Standard deviations were below 5%. (c) Erythritol concentration (given in mg/l cultivation broth) was measured by GC in cultivation broth samples after cell disruption. Standard deviations were obtained from measurements in triplicates. (d) Transcript analysis of *err1* (given as relative transcript ratio in logarithmic scale (lg)) was performed by qPCR using sar1 and act as genes for data normalization and levels always refer to Rut-C30 cultivated for 48 h (as indicated by an asterisk). Standard deviations were obtained from measurements in triplicates. Biological experiments (cultivations) were performed in duplicates. Click here for file
