# Supplementary material for: Erythritol production on wheat straw using Trichoderma reesei
Source: AMB Express. 2014 May 29;4:34. doi: 10.1186/s13568-014-0034-y (PMC4052684; doi:10.1186/s13568-014-0034-y)
Supplement: Additional file 1 — Figure S1. Cultivation of Rut-C30 and an err1 overexpression strain on wheat straw. The Rut-C30 (blue bars) and the err1 overexpression strain RPEC1 (red bars) were cultivated in bench-top bioreactors on pre-treated wheat straw. Samples were taken after 48 and 72 hours. (a) Sodium soluble protein concentration (given in g/l cultivation broth) was measured in triplicates in cultivation broth samples after cell disruption to indicate biomass formation. Standard deviations were below 5%. (b) Xylanase activity (given in U/l cultivation broth) was measured in triplicates in the cultivation supernatants. Standard deviations were below 5%. (c) Erythritol concentration (given in mg/l cultivation broth) was measured by GC in cultivation broth samples after cell disruption. Standard deviations were obtained from measurements in triplicates. (d) Transcript analysis of err1 (given as relative transcript ratio in logarithmic scale (lg)) was performed by qPCR using sar1 and act as genes for data normalization and levels always refer to Rut-C30 cultivated for 48 h (as indicated by an asterisk). Standard deviations were obtained from measurements in triplicates. Biological experiments (cultivations) were performed in duplicates. [file s13568-014-0034-y-S1.pdf]

Fig. S1

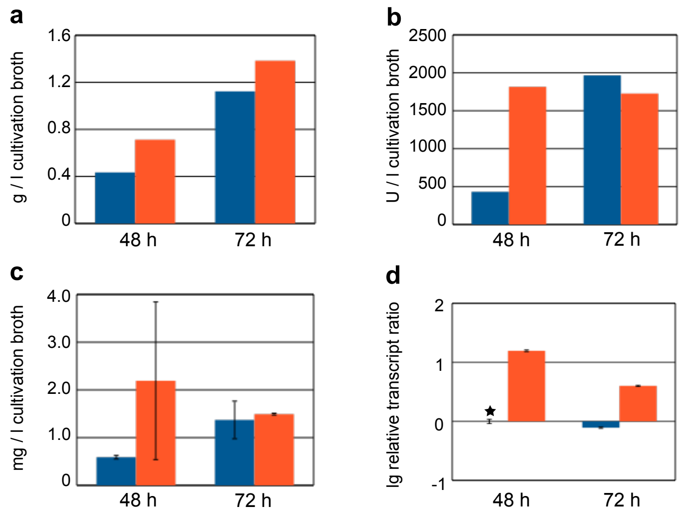

Figure S1 – Cultivation of Rut-C30 and an *err1* overexpression strain on wheat straw. The Rut-C30 (blue bars) and the *err1* overexpression strain RPEC1 (red bars) were cultivated in bench-top bioreactors on pre-treated wheat straw. Samples were taken after 48 and 72 hours. (a) Sodium soluble protein concentration (given in g/l cultivation broth) was measured in triplicates in cultivation broth samples after cell disruption to indicate biomass formation. Standard deviations were below 5 %. (b) Xylanase activity (given in U/l cultivation broth) was measured in triplicates in the cultivation supernatants. Standard deviations were below 5 %. (c) Erythritol concentration (given in mg/l cultivation broth) was measured by GC in cultivation broth samples after cell disruption. Standard deviations were obtained from measurements in triplicates. (d) Transcript analysis of *err1* (given as relative transcript ratio in logarithmic scale (lg)) was performed by qPCR using *sar1* and *act* as genes for data normalization and levels always refer to Rut-C30 cultivated for 48 h (as indicated by an asterisk). Standard deviations were obtained from measurements in triplicates. Biological experiments (cultivations) were performed in duplicates.
